# TANGO: A reliable, open-source, browser-based task to assess individual differences in gaze understanding in 3 to 5-year-old children and adults

**DOI:** 10.3758/s13428-023-02159-5

**Published:** 2023-07-10

**Authors:** Julia Christin Prein, Steven Kalinke, Daniel B. M. Haun, Manuel Bohn

**Affiliations:** 1 Department of Comparative Cultural Psychology, Max Planck Institute for Evolutionary Anthropology, Deutscher Platz 6, 04103 Leipzig, Germany; 2 Institute of Psychology, Leuphana University Lüneburg, Lüneburg, Germany

**Keywords:** Social cognition, Individual differences, Gaze cues, Cognitive development

## Abstract

Traditional measures of social cognition used in developmental research often lack satisfactory psychometric properties and are not designed to capture variation *between* individuals. Here, we present the TANGO (Task for Assessing iNdividual differences in Gaze understanding-Open); a brief (approx. 5–10min), reliable, open-source task to quantify individual differences in the understanding of gaze cues. Localizing the attentional focus of an agent is crucial in inferring their mental states, building common ground, and thus, supporting cooperation. Our interactive browser-based task works across devices and enables in-person and remote testing. The implemented spatial layout allows for discrete and continuous measures of participants’ click imprecision and is easily adaptable to different study requirements. Our task measures inter-individual differences in a child (*N* = 387) and an adult (*N* = 236) sample. Our two study versions and data collection modes yield comparable results that show substantial developmental gains: the older children are, the more accurately they locate the target. High internal consistency and test–retest reliability estimates underline that the captured variation is systematic. Associations with social-environmental factors and language skills speak to the validity of the task. This work shows a promising way forward in studying individual differences in social cognition and will help us explore the structure and development of our core social-cognitive processes in greater detail.

**Supplementary Information:**

The online version contains supplementary material available at 10.3758/s13428-023-02159-5.

## Introduction

Social cognition–representing and reasoning about an agent’s perspectives, knowledge states, intentions, beliefs, and preferences to explain and predict their behavior – is among the most-studied phenomena in developmental research. In recent decades, much progress has been made in determining the average age at which a specific social-cognitive ability emerges in development (Gopnik & Slaughter, 1991; Peterson et al., 2012; Rakoczy, 2022; Wellman et al., 2001; Wellman & Liu, 2004). Yet, there are always individual differences. Identifying variability in social-cognitive abilities and factors influencing their development is vital in theory building (e.g., to test causal predictions) and designing interventions (Happé et al., 2017; Kidd et al., 2018; Lecce et al., 2014; Mundy et al., 2007; Underwood, 1975).

Numerous studies have already examined individual differences in social cognition (for an overview, see Hughes & Devine, 2015; Slaughter, 2015). The most common, recurring research questions are concerned with the developmental sequence of social-cognitive abilities (e.g., Wellman & Liu, 2004), and which factors drive the development of social cognition (Devine & Hughes, 2018; Gola, 2012). For example, Okumura and colleagues asked how early gaze-following and object processing relate to later language development (Okumura et al., 2017). In general, individual differences studies often focus on the relationship between social-cognitive abilities and: (1) family influences, (2) other cognitive constructs, and (3) social behavioral outcomes (for an overview, see Slaughter and Repacholi, 2003). Studies on social-cognitive abilities and family influences include the effect of parenting practices (for a review, see Pavarini et al., 2013), attachment quality (e.g., Astor et al., 2020), mental state talk (Gola, 2012; Hughes et al., 2011; Lecce et al., 2014), and family background as parental education, occupation, sibling interaction and childcare (Bulgarelli & Molina, 2016; Cutting & Dunn, 1999; Dunn et al., 1991). Another group of individual differences studies focuses on the interplay of social and physical cognition (Herrmann et al., 2010), executive functions (Benson﻿ et al., 2013; Buttelmann et al., 2022; Carlson & Moses, 2001; Carlson et al., 2004; Hughes & Ensor, 2007), and language abilities (McEwen et al., 2007; Milligan et al., 2007; Okumura et al., 2017). Studies on social behavioral outcomes measured the interplay of social cognition and prosociality (for a review, see Imuta et al., 2016; Walker, 2005), stereotypes, resource allocations (Rizzo & Killen, 2018), and moral intentions (Sodian et al., 2016).

However, developmental psychologists are frequently surprised to find minor or no association between measures of social cognition that are thought to be theoretically related – cross-sectionally and/or longitudinally (e.g., Poulin-Dubois et al., 2023; Sodian, 2023; Sodian et al., 2016). This might be because traditional measures of social cognition are not designed to capture variation *between* children: they often rely on low trial numbers, small sample sizes, and dichotomous measures. A recent review showed that many studies on social cognition measures failed to report relevant psychometric properties at all (Beaudoin et al., 2020) or – when they did – showed mixed results on test–retest reliability (Hughes et al., 2000; Mayes et al., 1996).

To give an example: the most commonly applied prototypical measure for social cognition is the change-of-location false belief task (Baron-Cohen et al., 1985; Wimmer & Perner, 1983). Here, children watch a short sequence of events (often acted out or narrated by the experimenters). A doll called Sally puts her marble into a basket. After Sally leaves the scene, a second doll named Anne takes the marble and moves it into a box. Participants then get asked where Sally will look for her marble once she returns. The outcome measures false belief understanding in a dichotomous way: children pass the task if they take the protagonist’s epistemic state into account and answer that she will look into the basket. Many years of research utilizing these verbal change-of-location tasks suggest that children develop belief-representing abilities at four to five years of age (for a review, see Wellman et al., 2001). Several cross-cultural studies supported this evidence (Barrett et al., 2013; Callaghan et al., 2005; cf. Mayer & Träuble, 2015).

However, from this age onwards, the change-of-location task shows ceiling effects and has very limited diagnostic value (Repacholi, 2003). Thus, this task seems well suited to track a particular group-level developmental transition, yet it fails to capture individual differences (cf. “reliability paradox,” Hedge et al., 2018). As Wellman (2012) put it, “it’s really only passing/failing one sort of understanding averaged across age” (p. 317). This has profound implications for what studies on individual differences using this task (or others) can show. Poor measurement of social cognition on an individual level is likely to conceal relations between different aspects of cognition and may obscure developmental change. For example, Sodian et al. (2016) neither found a correlation between two moral Theory of Mind False Belief and Intention tasks at 60 months, nor a relationship between these two factors and implicit False Belief understanding at 18 months.

The “Sandbox task” is one of the few tasks that attempt to overcome these methodological challenges (Begeer et al., 2012; Bernstein et al., 2011; Coburn et al., 2015; Mahy et al., 2017; Sommerville et al., 2013). This continuous FB task measures the degree to which the estimate of another’s belief is biased by one’s own knowledge. Recent work questions the interpretation of this measure (Samuel et al., 2018a, b): it is unclear whether a smaller egocentric bias can be directly translated into a better mental state reasoning ability. Another evaluation criterion should, therefore, be whether a task captures meaningful variability in performance; that is, differences in test scores should correspond to differences in the social-cognitive ability in question.

Thus, developmental psychology faces a dilemma: many research questions rely on measuring individuals’ development, yet, there is a lack of tasks to measure these individual differences reliably. To capture the emergence of social-cognitive abilities and their relation to social factors in greater precision and detail, we must consequently address the methodological limitations of existing study designs (Hughes et al., 2011; Hughes & Leekam, 2004).

Schaafsma et al., (2015) compiled a “wish list” for new social-cognitive paradigms. They advocated for parametric – instead of dichotomous – measures covering proficiency as a range, avoiding floor and ceiling effects, and showing satisfactory test–retest reliability estimates (see also Beaudoin et al., 2020; Hughes & Devine, 2015). New tasks should capture variation across age groups, including older children and adults (Repacholi and Slaughter, 2003). Another goal in creating new tasks should be to focus on the “face value”: measures should probe the underlying social-cognitive ability as straight-forward and directly as possible. Keeping task demands minimal is also beneficial for using the paradigm in a variety of different cultural, clinical, and demographic contexts (Molleman et al., 2019). The task should serve as a proxy for behavior as it appears in the real world and should be validated in relation to real-world experiences (Repacholi and Slaughter, 2003).

### A new measure of gaze understanding

Our goal was to design a new measure of social cognition that captures individual differences across age groups in a systematic, reliable, and valid way. We focused on a fundamental ability implicated in many social-cognitive reasoning processes: gaze understanding – the ability to locate and use the attentional focus of an agent. The first component of this ability is often termed gaze following – turning one’s eyes in the same direction as the gaze of another agent – and has been studied intensively (Astor et al., 2021; Byers-Heinlein et al., 2021; Coelho et al., 2006; Del Bianco et al., 2019; Frischen et al., 2007; Hernik & Broesch, 2019; Itakura & Tanaka, 1998; Lee et al., 1998; Moore, 2008; Shepherd, 2010; Tomasello et al., 2007). In our definition, gaze understanding goes one step further by including the *acting on the gaze-cued location* – therefore, using the available social information to guide one’s behavior as needed in real-life conditions.

Following an agent’s gaze provides insights into their intentions, thoughts, and feelings by acting as a “front end ability” (Brooks & Meltzoff, 2005, p. 535). Gaze is integral for many more sophisticated social-cognitive abilities, for example, inferences about knowledge states. As such, the eyes have been regarded as a “window into the mind” (Shepherd, 2010). Monitoring another’s attention also supports building a common ground, which is important for action coordination and cooperative social interactions (Bohn & Köymen, 2018; Tomasello et al., 2007). In addition, gaze and language development seem to be related (Brooks & Meltzoff, 2005). Gaze facilitates word learning by helping to identify the referent of a new word and has been regarded as a crucial signal of nonverbal communication (Hernik & Broesch, 2019; Macdonald & Tatler, 2013).

While the emergence of gaze following has been well established, less is known about the developmental trajectory throughout childhood and adolescence. One possibility is that our social-cognitive ability in question is fully developed once emerged in infancy. However, many cognitive abilities continue to develop beyond early childhood (e.g., Gathercole et al., 2004 for working memory; Raviv & Arnon, 2018 for visual statistical learning). Therefore, children could potentially improve in understanding gaze, fine-tuning the performance of the already existing skill. Consequently, we aimed to assess the differentiation of the ability to understand gaze. Our goal was *not* to establish the youngest age at which children understand gaze cues. Rather, we wanted to examine how that ability changes with age. To accurately measure developmental change, we were interested in capturing individual variability.

To address the psychometric shortcoming of earlier work, we implemented the following design features: First, we used a continuous measure which allowed us to capture fine-grained individual differences at different ages. Second, we designed short trials that facilitate more than a dozen replicates per subject. The result is more precise individual-level estimates. Third, we systematically investigated the psychometric properties of the new task.

Designing this task required a new testing infrastructure. We designed the task as an interactive web application. Previous research has successfully used online study implementations that compare well to in-person data collection (Bohn et al., 2021a, b; Frank et al., 2016). This greatly increased the flexibility with which we could modify the stimuli on a trial-by-trial basis. Furthermore, because the task is largely self-contained, it is much more controlled and standardized. Most importantly, it makes the task portable: testing is possible in-person using tablets but also remotely via the internet (no installation needed). As such, it provides a solid basis to study individual differences in gaze understanding across ages at scale. We make the task and its source code openly accessible for other researchers to use and modify.

## Task design

### Implementation

The code is open-source (https://github.com/ccp-eva/tango-demo), and a live demo version can be found under: https://ccp-odc.eva.mpg.de/tango-demo/.

The web app was developed using JavaScript, HTML5, CSS, and PHP. For stimulus presentation, a scalable vector graphic (SVG) composition was parsed. This way, the composition scales according to the user’s viewport without loss of quality while keeping the aspect ratio and relative object positions constant. Furthermore, SVGs allow us to define all composite parts of the scene (e.g., pupil of the agent) individually. This is needed for precisely calculating the exact pupil and target locations and sizes. Additionally, it makes it easy to adjust the stimuli and, for example, add another agent to the scene. The web app generates two file types: (1) a text file (.json) containing metadata, trial specifications, and participants’ click responses, and (2) a video file (.webm) of the participant’s webcam recording. These files can either be sent to a server or downloaded to the local device. Personalized links can be created by passing on URL parameters.

### Stimuli

**Fig. 1:**
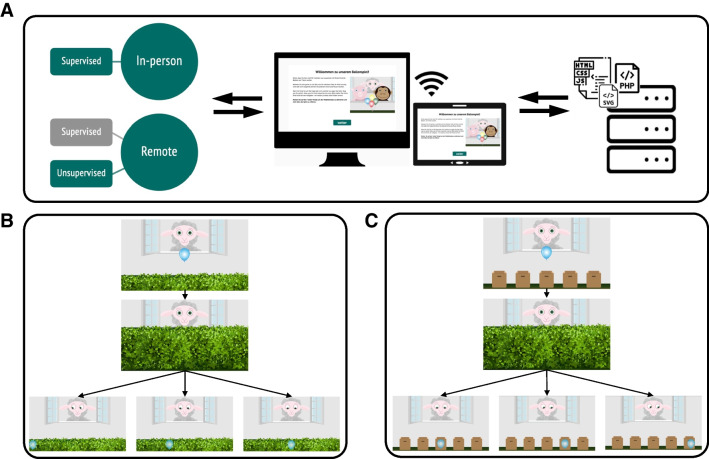
Study setup. **A** Infrastructure for online testing. (i) Subjects aged 3 to 99+ can participate. Data collection can take place anywhere: online, in kindergartens, or in research labs. (ii) The task is presented as a website that works across devices. (iii) The scripts for the website and the recorded data are stored on secure in-house servers. **B** Hedge version (continuous) of the TANGO. (i) The agent stands in a window with the target in front of them. (ii) A hedge grows and covers the target. (iii) The target falls to a random location on the ground. The agent’s eyes track the movement of the target. Three exemplary target locations are shown to depict how indicative the agent’s gaze cues are in determining the target’s location. The transparent target is only shown for an illustrative purpose (not visible during the test). **C** Box version (discrete) of the TANGO. Number of boxes (min. 1; max. 8) as potential hiding locations can be set according to the researcher’s need

Our newly implemented task asks children and adults to search for a balloon. The events proceed as follows (see Fig. 1B and C). An animated agent (a sheep, monkey, or pig) looks out of a window of a house. A balloon (i.e., target; blue, green, yellow, or red) is located in front of them. The target then falls to the ground. At all times, the agent’s gaze tracks the movement of the target: the pupils and iris move so that their center aligns with the center of the target. While the distance of the target’s flight depends on the final location, the target moves at a constant speed. Participants are then asked to locate the target: they respond by touching or clicking on the screen. Visual access to the target’s true location is manipulated by a hedge. Participants either have full, partial, or no visual access to the true target location. When partial or no information about the target location is accessible, participants are expected to use the agent’s gaze as a cue.

To keep participants engaged and interested, the presentation of events is accompanied by cartoon-like effects. Each trial starts with an attention-getter: an eye-blinking sound plays while the pupils and iris of the agent enlarge (increase to 130%) and change in opacity (decrease to 75%) for 0.3 s. The landing of the target is accompanied by a tapping sound. Once the target landed, the instructor’s voice asked “Where is the balloon?”. To confirm the participant’s click, a short plop sound plays, and a small orange circle appears at the location of choice. Participants do not receive differential feedback so that learning effects are reduced, and trials stay comparable across the sample. If no response is registered within 5 s after the target landed, an audio prompt reminds the participant to respond.

### Trials

Trials differ in the amount of visual access that participants have to the final target position. Before the test trials start, participants complete four training trials during which they familiarize themselves with touching the screen. In the first training trial, participants have full visual access to the target flight and the target’s end location and are simply asked to click on the visible balloon. In the second and third training trials, participants have partial access: they witness the target flight but cannot see the target’s end location. They are then asked to click on the hidden balloon, i.e., the location where they saw the target land. In test trials, participants have no visual access to the target flight or the end location. Participants are expected to use the agent’s gaze as a cue to locate the target. The first trial of each type comprises a voice-over description of the presented events. The audio descriptions explicitly state that the agent is always looking at the target (see Supplements for audio script). After the four training trials, participants receive 15 test trials. The complete sequence of four training trials and 15 test trials can be easily completed within 5–10 min.

### Study versions

We designed two study versions that differ in the target’s final hiding place and, consequently, in the outcome measure: a *hedge version* (continuous) and a *box version* (discrete). Both versions use the same first training trial and then differ in the consecutive training and test trials. In the hedge version, participants have to indicate their estimated target location directly on a hedge. Here, the dependent variable is imprecision, which is defined as the absolute difference between the target center and the *x* coordinate of the participant’s click. In the box version, the target lands in a box, and participants are asked to click on the box that hides the target. Researchers can choose how many boxes are shown: one up to eight boxes can be displayed as potential hiding locations. Here, we use a categorical outcome (i.e., which box was clicked) to calculate the proportion of correct responses. Note that in the test trials of both versions, the target flight is covered by a hedge. In the hedge version, the hedge then shrinks to a minimum height required to cover the target’s end location. In the box version, the hedge shrinks completely. The boxes then hide the target’s final destination (see Fig. 1B and C).

### Randomization

All agents and target colors appear equally often and are not repeated in more than two consecutive trials. The randomization of the target end location depends on the study version. In the hedge version, the full width of the screen is divided into ten bins. Exact coordinates within each bin are then randomly generated. In the box version, the target randomly lands in one of the boxes. As with agent and color choice, each bin/box occurs equally often and can only occur twice in a row.

## Individual differences

Our first aim was to assess whether the TANGO captures inter-individual variation in a child and adult sample. Furthermore, we were interested in whether and how the data collection mode (in-person vs. remote) influences responses. Since we expected a greater difference in responses between the two data collection modes for children, the analysis of data collection mode was restricted to a child sample.

Task design, data collection, and sample sizes were pre-registered: https://osf.io/snju6 (child sample) and https://osf.io/r3bhn (adult sample). The analyses reported here were not pre-registered but followed the structure of the ones specified in the above pre-registrations (see Footnotes for deviations). The additional analyses mentioned in the pre-registrations (e.g., computational model) address separate research questions (e.g., process-level account of gaze understanding) and will be reported elsewhere. In this paper, we focus on the methodological and psychometric aspects of our task.

The study design and procedure obtained ethical clearance by the MPG Ethics commission Munich, Germany, falling under a packaged ethics application (Appl. No. 2021_45), and was approved by an internal ethics committee at the Max Planck Institute for Evolutionary Anthropology. The research adheres to the legal requirements of psychological research with children in Germany.

Participants were equally distributed across the two study versions. Data were collected between May and October 2021.

### Participants

We collected data from an in-person child sample, a remote child sample, and a remote adult sample. In-person testing with children took place in kindergartens in Leipzig, Germany. The in-person child sample consisted of 120 children, including 40 3-year-olds (mean = 41.45 months, SD = 3.85, range = 36–47, 22 girls), 40 4-year-olds (mean = 54.60 months, SD = 3.10, range = 48–59, 19 girls), and 40 5-year-olds (mean = 66.95 months, SD = 3.39, range = 60–71, 22 girls).

We pre-registered the replacement for participants that finished fewer than four test trials. This was not the case for any participant. One child stopped participation after 12 test trials but was included in the sample due to the pre-registered replacement rule. Two additional participants were recruited but not included in the study because the participant did not feel comfortable interacting with the tablet alone (*n* = 1), or due to an originally miscalculated age of the child (*n* = 1).

For our remote child sample, we recruited families via an internal database of children living in Leipzig, Germany, whose parents volunteered to participate in child development studies and who indicated an interest in online studies. Families received an email with a short study description and a personalized link. If they had not participated in the study within 2 weeks, they received a reminder via e-mail. The response rate to invitations after the reminder was ~ 50%.

The remote child sample included 147 children, including 45 3-year-olds (mean = 42.62 months, SD = 3.35, range = 36–47, 14 girls), 47 4-year-olds (mean = 52.64 months, SD = 3.40, range = 48–59, 25 girls), and 55 5-year-olds (mean = 65.11 months, SD = 3.77, range = 60–71, 27 girls). Of these, three families participated twice. In these cases, we only kept the data sets from the first participation.

Four additional participants were recruited but not included in the study because they were already part of the in-person kindergarten sample (*n* = 3), or because of unknown age (*n* = 1).

Please note that we did not collect participant-specific demographics. In the following, we aim to provide context and generalizations based on the broader community and the larger pool of potential participants. Children in our sample grow up in an industrialized, urban Central-European context in a city with approximately 600,000 inhabitants. They often live in nuclear two-generational families with few household members. Information on socioeconomic status was not formally recorded, although the majority of families come from mixed, mainly mid to high socioeconomic backgrounds with high levels of parental education. The median individual monthly net income in the year 2021 was ~ 1,600€ for the city of Leipzig.

Adults were recruited via *Prolific* (Palan & Schitter, 2018). *Prolific* is an online participant recruitment service with a predominantly European and US–American subject pool. One hundred English speakers with an average age of 31.34 years (SD = 10.77, range = 18–63, 64 females) were included. Participants live in a variety of different countries: the UK, Italy, Spain, Poland, Netherlands, Canada, Australia, Ireland, South Africa, Norway, Portugal, France, Austria, Finland, Greece, Germany, the U.S., Mexico, Chile, Iceland, New Zealand, Czech Republic, Hungary, Latvia, and Switzerland. In this sample, most participants resided in the United Kingdom (*n* = 47), South Africa (*n* = 8), and Portugal (*n* = 6). Additional detailed information can be found in the data set online. For completing the study, subjects were paid above the fixed minimum wage (on average £10.00 per hour; see Supplements for further detail).

### Procedure

Children in our in-person sample were tested on a tablet in a quiet room in their kindergarten. An experimenter guided the child through the study.

Children in the remote sample received a personalized link to the study website, and families could participate at any time or location. At the beginning of the online study, families were invited to enter our “virtual institute”. We welcomed them with a short introductory video of the study leader, describing the research background and further procedure. Then, caregivers were informed about data security and were asked for their informed consent. They were asked to enable the sound and seat their child centrally in front of their device. Before the study started, families were instructed on how to set up their webcam and enable the recording permissions. We stressed that caregivers should not help their children. Study participation was video recorded whenever possible in order to ensure that the children themselves generated the answers. Depending on the participant’s device, the website automatically presented the hedge or box version of the study. For families that used a tablet with a touchscreen, the hedge version was shown. Here, children could directly click on the touchscreen to indicate where the target is. For families that used a computer without a touchscreen, the website presented the box version of the task. We assumed that younger children in our sample would not be acquainted with using a computer mouse. Therefore, we asked children to point to the screen, while caregivers were asked to act as the “digital finger” of their children and click on the indicated box.

All participants received 15 test trials. In the box version, we decided to adjust the task difficulty according to the sample: children were presented with five boxes, while adults were presented with eight boxes as possible target locations.

### Analysis

All test trials without voice-over descriptions were included in our analyses. We ran all analyses in R version 4.3.0 (2023-04-21) (R Core Team, 2022). Regression models were fitted as Bayesian generalized linear mixed models (GLMMs) with default priors for all analyses, using the function brm from the package brms (Bürkner, 2017, 2018).

To estimate the developmental trajectory of gaze understanding and the effect of data collection mode, we fit a GLMM predicting the task performance in each trial by age (in months, z-transformed) and data collection mode (reference category: in-person supervised). The model included random intercepts for each participant and symmetric target position, and a random slope for symmetric target position within participants (model notation in R: performance ~ age + datacollection + symmetricPosition + trialNr + (1 + symmetricPosition + trialNr | subjID)).^1^

Here, symmetricPosition refers to the absolute distance from the stimulus center (i.e., smaller value meaning more central target position). We expected that trials could differ in their difficulty depending on the target centrality and that these item effects could vary between participants.

For the hedge version, performance was defined as the absolute click distance between the target center and the click x coordinate, scaled according to target widths, and modeled by a lognormal distribution. For the box version, the model predicted correct responses (0/1) using a Bernoulli distribution with a logit link function. We inspected the posterior distribution (mean and 95% credible interval (CrI)) for the age and data collection estimates.

### Results

Children showed nearly perfect precision in the first training trial. As visual access to the target location decreased in the subsequent training trials, imprecision levels increased (see Supplements). Within test trials, children’s imprecision levels did not vary as a function of trial number. We take this as evidence that (A) children were comfortable touching the screen, (B) children understood the task instructions insofar as they aimed at locating the target, and (C) our experimental design successfully manipulated task difficulty.

**Fig. 2:**
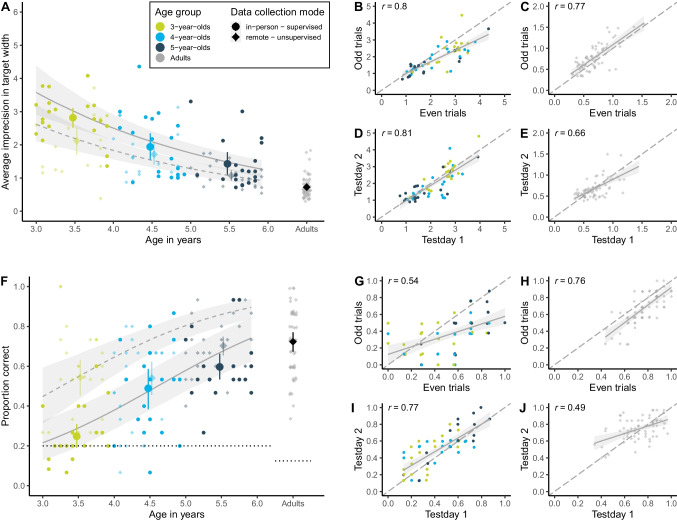
Measuring inter-individual variation. **A** Developmental trajectory in the continuous hedge version. Performance is measured as imprecision, i.e., the absolute distance between the target’s center and the participant’s click (averaged across trials). The unit of imprecision is counted in the width of the target, i.e., a participant with imprecision of 1 clicked on average one target width to the left or right of the true target center. **B** Internal consistency (odd-even split) in hedge child sample. **C** Internal consistency in hedge adult sample. **D** Test–retest reliability in hedge child sample. **E** Test–retest reliability in hedge adult sample. **F** Developmental trajectory in the discrete box version. Performance is measured as the proportion of correct responses, i.e., how many times the participant clicked on the box that contained the target. The *dotted black line* shows the level of performance expected by chance (for child sample 20%, i.e., one out of five boxes; for adult sample 12.5%, i.e., one out of eight boxes). **G** Internal consistency (odd-even split) in box child sample. **H** Internal consistency in box adult sample. **I** Test–retest reliability in box child sample. **J** Test–retest reliability in box adult sample. For (A) and (F), regression lines show the predicted developmental trajectories (with 95% CrI) based on GLMMs, with the *line type* indicating the data collection mode. *Large points* with 95% CI (based on non-parametric bootstrap) represent performance means by age group (binned by year). *Small points* show the mean performance for each subject averaged across trials. For adult data in (A) and (F), we added minimal horizontal and vertical noise to avoid overplotting. The *shape of data points* represents data collection mode: *opaque circles* for in-person supervised data collection and *translucent diamonds* for remote unsupervised data collection. The *color of data points* denotes age group. For (B–E) and (G–J), *regression lines* with 95% CI show smooth conditional mean based on a linear model (generalized linear model for box version), with *Pearson*’s correlation coefficient *r*

We found a strong developmental effect: with increasing age, participants got more accurate in locating the target. In the hedge version, children’s click imprecision decreased with age, while in the box version, the proportion of correct responses increased (see Fig. 2A and F). Most participants in the box version performed above chance level. By the end of their sixth year of life, children came close to the adult’s proficiency level. Most importantly, however, we found substantial inter-individual variation across study versions and age groups. For example, some 3-year-olds were more precise in their responses than some 5-year-olds. Even though variation is smaller, we could even find inter-individual differences in the adult sample.

As Fig. 2A and F show, our remotely collected child data resembled the data from the kindergarten sample. We found evidence that responses of children participating remotely were slightly more precise. This difference was mainly driven by the younger participants and was especially prominent in the box version of the task. It is conceivable that caregivers were especially prone to influence the behavior of younger children. In the box version, caregivers might have had more opportunities to interfere since they carried out the clicking for their children.^2^

Our GLMM analysis corroborated the visual inspection of the data: in the hedge version, the estimates for age ($\beta$ = – 0.32; 95% CrI [– 0.41; – 0.23]) and data collection mode – 0.31 (95% CrI [– 0.48; – 0.14]) were negative and reliably different from zero. In the box version, the estimate of age ($\beta$ = 0.68 (95% CrI [0.44; 0.93]) and the estimate of data collection mode ($\beta$ = 1.10 (95% CrI [0.66; 1.56]) were positive and reliably different from zero. Note that even though confidence intervals from the data collection estimates were wide, the effect was positive and reliably different from zero in that our remote sample performed more accurately than our in-person sample.

There was no effect of trial number (hedge version: $\beta$ = 0.00; 95% CrI [– 0.02; 0.01]; box version: $\beta$ = – 0.02; 95% CrI [– 0.05; 0.01). However, trials differed in difficulty depending on where the target landed (hedge version: $\beta$ = 0.47; 95% CrI [0.40; 0.54]; box version: $\beta$ = – 1.59; 95% CrI [– 1.88; – 1.31). When the target landed closer to the center of the screen, participants were more accurate in locating it.

### Discussion

Our task measured inter-individual differences in both children and adults; that is, we found substantial variation in individuals across age groups. For example, some 3-year-olds showed greater precision levels than some 5-year-olds. This holds across both study versions. However, due to the continuous study design, the hedge version was able to capture more fine-grained differences in individual performance. We see substantial developmental gains: with increasing age, participants became on average more and more precise in locating the target. The 5-year-olds reached a proficiency level close to the adults’ level. For neither study version nor age group did we find any floor or ceiling effects. The presentation as a web app with cartoon-like features kept children interested and motivated throughout the 15 test trials. Furthermore, we found a comparable developmental trajectory for an unsupervised remote child sample. This illustrates the flexibility of the task design.

## Internal consistency and test–retest reliability

As a next step, we aimed to investigate whether the variation that we captured with the TANGO is reliable. We assessed internal consistency (as split-half reliability) and test–retest reliability. Task procedure, data collection, and sample sizes were pre-registered (https://osf.io/xqm73 for the child sample and https://osf.io/nu62m for the adult sample). Participants were equally distributed across the two study versions. Data was collected between July 2021 and June 2022.

The study design and procedure obtained ethical clearance by the MPG Ethics commission Munich, Germany, falling under a packaged ethics application (Appl. No. 2021_45), and was approved by an internal ethics committee at the Max Planck Institute for Evolutionary Anthropology. The research adheres to the legal requirements of psychological research with children in Germany.

### Participants

Participants were recruited in the same way as in the previous study. The child sample consisted of 120 children, including 41 3-year-olds (mean = 42.34 months, SD = 3.10, range = 37–47, 20 girls), 41 4-year-olds (mean = 53.76 months, SD = 3.15, range = 48–59, 21 girls), and 38 5-year-olds (mean = 66.05 months, SD = 3.40, range = 60–71, 19 girls).

Additional 65 children were recruited but not included in the analysis due to absence on the second test day (*n* = 49), canceled testing because of COVID-19 cases in the kindergarten (*n* = 7), children did not want to participate a second time (*n* = 5), children already participated in the first data collection round and were included in the above-mentioned *Individual Differences* sample (*n* = 3), or children did not understand the task instructions (*n* = 1; manifested in too early clicking in the training trials while the instructions were still playing, and no clicking by themselves in the test trials). Two additional children were recruited for the first day (as backup) in case another child would be absent on the second test day. Similar to our first study, we did not collect participant-specific demographics. For a community-based description of our participant pool, see Participant section of the first study.

As in our first study, adults were recruited via *Prolific* (Palan & Schitter, 2018). The adult sample included 136 English speakers with an average age of 25.73 years (SD = 8.09, range = 18–71, 87 females; see Supplements for further details). Most participants resided in South Africa (*n* = 48), the United Kingdom (*n* = 19), and the United States (*n* = 14). See Supplements and the available online data set for more detailed information.

### Procedure

We applied the same procedure as in the first study, with the following differences. Participants completed the study twice, with a delay of 14 ± 3 days. The target locations, as well as the succession of agents and target colors, were randomized once and then held constant across participants. The child sample received 15 test trials. In the hedge version, each bin occurred once, making up ten of the test trials. For the remaining five test trials, we repeated one out of two adjacent bins (i.e., randomly chose between bins 1 & 2, bins 3 & 4, etc.). In the box version, we ensured that each of the five boxes occurred exactly three times during test trials. Adults in the hedge version received 30 test trials, each of the ten bins occurring exactly three times. Adults in the box version received 32 test trials, with each of the eight boxes occurring exactly four times. For the four training trials, we repeated a fixed order of random bins/boxes. For the adult sample, we decided to increase the number of trials in order to get more accurate reliability estimates. Trial numbers were multipliers of the possible target locations and therefore differed between hedge and box versions. For the child sample, we stuck to the same number of trials to not risk higher attrition rates.

### Analysis

We assessed reliability in two ways. First, we focused on internal consistency by calculating split-half reliability coefficients.^3^ For each subject, trials were split into odd and even trials. Performance was aggregated and then correlated using *Pearson* correlation coefficients. For this, we used the data of the first test day. Performance was defined according to each study version: in the hedge version, performance referred to the mean absolute difference between the target center and the click coordinate, scaled according to target widths; in the box version, we computed the mean proportion of correct choices.

﻿Pronk et al., (2022) recently compared various methods for computing split-half reliability that differ in how the trials are split into parts and whether they are combined with stratification by task design. To compare our traditional approach of a simple odd-even split, we additionally calculated split-half reliability estimates using first-second, odd-even, permutated, and Monte Carlo splits without and with stratification by target position. First-second and odd-even splits belong to single sample methods since each participant has a single pair of performance scores, while permutated (without replacement) and Monte Carlo (with replacement) splits make use of resampling. Analyses were run using the function by_split from the splithalfr package (Pronk et al., 2021).

Second, we assessed test–retest reliability. We calculated performance scores (depending on the study version as described above) for each participant in each test session and correlated them using *Pearson* correlation coefficients. Furthermore, for our child sample, we report an age-corrected correlation between the two test days using a GLMM-based approach (Rouder & Haaf, 2019). We fit trial-by-trial data with a fixed effect of age, a random intercept for each subject, and a random slope for test day (model notation in R: performance ~ age + (0 + reliday | subjID)). For the hedge version, performance was modeled by a lognormal distribution, while the model for the box version used a Bernoulli distribution with a logit link function. The model computes a correlation between the participant-specific estimates for each test day. This can be interpreted as the test–retest reliability. By using this approach, we do not need to compromise on data aggregation and, therefore, loss of information. Since the model uses hierarchical shrinkage, we obtain regularized, more accurate person-specific estimates. Most importantly, the model includes age as a fixed effect. The correlation between the two person-specific estimates is consequently the age-independent estimate for test–retest reliability. This rules out the possibility that a high correlation between test days arises from domain-general cognitive development instead of study-specific inter-individual differences. A high correlation between our participant-specific model estimates would indicate a high association between test days.

### Results

We found that the TANGO measured systematic variation: split-half and test–retest reliability was medium to high. For internal consistency, we show traditional odd-even splits on our data and the corresponding *Pearson* correlation coefficients in Fig. 2B, C, G, and H.

**Fig. 3:**
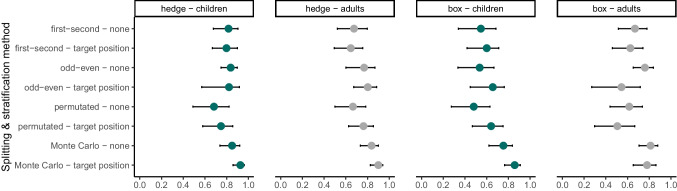
Internal consistency. Reliability coefficients per splitting method, stratification level, study version, and age group. *Error bars* show the 95% confidence intervals of the coefficient estimates, calculated with the function by_split from the splithalfr package (Pronk et al., 2021)

Figure 3 compares split-half reliability coefficients by splitting and stratification method (Pronk et al., 2021). In the hedge version, the split-half reliability coefficients ranged from 0.65 to 0.93. In the box version, split-half reliability coefficients ranged from 0.48 to 0.86. Similar to the results of Pronk et al. (2021), we found that more robust splitting methods that are less prone to task design or time confounds yielded higher reliability coefficients. In most cases, stratifying by target position led to similar or even higher estimates compared to no stratification. As expected, we found higher coefficients for the samples with higher variation, i.e., for our continuous hedge version of the task.

For test–retest reliability, we show the association between raw performance scores of the two test days and corresponding *Pearson* correlation coefficients in Fig. 2D, E, I and J.^4^ See Supplements for reliability estimates by age group.

The age-corrected, GLMM-based retest reliabilities for children yielded similar results. In the hedge version, the correlation between test days was 0.89 (95% CrI [0.64;1.00]). In the box version, the correlation between test days was 0.91 (95% CrI [0.70;1.00]).

For both study versions, reliability estimates based on the GLMM approach were higher than the *Pearson* correlations. The GLMM-based estimates are less noisy due to the fact that the model uses all available information (e.g., participant age) and does not rely on data aggregation across trials.

### Discussion

Our results indicated that the measured variation was systematic. As expected, the continuous measure of the hedge version yielded higher reliability estimates than the discrete box version. For children, the model-based reliability estimates showed that the task did capture individual differences even when correcting for age. This corroborates what we already saw in Fig. 2: there was a clear overlap between age groups, indicating that age is predictive of performance for the mean but is not the main source of individual differences.

## Validity

After having probed our new testing infrastructure and the psychometric properties of the TANGO, we aimed at establishing its validity. One way to assess validity is to correlate the social-cognitive ability in question to concepts that are thought to be theoretically related. Social cognition is often described as developing in response to social interaction (Devine & Hughes, 2018; Hughes & Leekam, 2004). It is assumed that opportunities to play, communicate and argue with peers help children to understand the human mind. Therefore, many studies link social cognition to opportunities for social interaction captured in demographic variables such as parent–child interaction quality and quantity, mental state talk, and center-based childcare (Bulgarelli & Molina, 2016; Dunn et al., 1991; Pavarini et al., 2013). In particular, family constellation, the number and age of siblings, and their interaction have been linked to social cognition (Cassidy et al., 2005; Dunn et al., 1991; Perner et al., 1994; Peterson, 2000; Zhang et al., 2021).

**Fig. 4:**
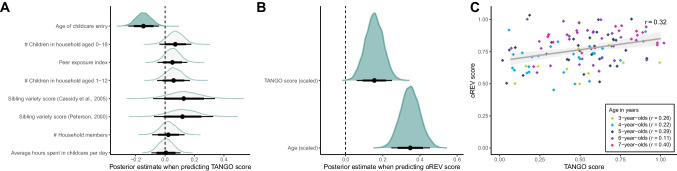
Validity of the TANGO. **A** Influence of social-environmental factors on gaze understanding. **B** Influence of gaze understanding on receptive vocabulary. For (A) and (B), the graphs show the posterior distributions for the respective predictor of each model. *Filled green density curves* show that adding the respective predictor improved the model fit compared to the null model. *Black dots* represent means, *thicker black lines* 80% CrI and *thinner black lines* 95% CrI. The oREV score is the proportion of correctly selected pictures in the receptive vocabulary task. Similarly, the TANGO score refers to proportion of correctly located targets (see Supplements for further detail). **C** Influence of gaze understanding on receptive vocabulary by age. The *regression line* with 95% CI shows a smooth conditional mean based on a generalized linear model, with *Pearson*’s correlation coefficient *r*. *Dots* show the mean performance for each subject averaged across trials with minimal horizontal and vertical noise added to avoid overplotting. The *color of dots* denotes age group

To assess such external validity for the TANGO, we handed out a brief demographic questionnaire to families of our kindergarten and online child sample and asked for (1) the total number of household members, (2) the number of children, (3) age of the other children, (4) whether the child was in daycare, and if yes, (5) since when and (6) for how long on an average day. 109 families filled out the questionnaire and were included in the analysis. We used parents’ responses to construct different scores suggested in the literature (Cassidy et al., 2005; Peterson, 2000), capturing aspects of children’s opportunities for social interaction with adults and peers. Only the predictor “age of childcare entry” improved the model fit compared to the null model (see Fig. 4A; for model comparisons, see Supplements): the older the children were when entering childcare, the less likely they were to correctly use the available gaze cue. Figure 4A shows that all other predictor scores were positively linked to gaze understanding. Effect sizes were probably influenced by the lack of variance in the predictors: variables like household size and number of siblings typically vary very little among German households (see Supplements for distribution characteristics of the predictors). Albeit the effects were weak, they are consistent with the literature.

In addition, children’s sensitivity to gaze has been linked to language acquisition (Brooks & Meltzoff, 2005; Del Bianco et al., 2019; Okumura et al., 2017). Discovering the attentional focus of your counterpart is thought to facilitate word learning, for example by identifying the referent of a new word (Tomasello, 2003). For 117 children, we also collected data with a receptive vocabulary test (oREV; Bohn et al., 2023) approximately 6 months (mean = 0.52 years, SD = 0.08, range = 0.06–0.80) after their participation in the TANGO. In the oREV task, children are shown four pictures (see Supplements for further detail) and hear a verbal prompt asking them to select one of the pictures. The oREV score is the proportion of correctly selected pictures. We found a substantial relationship between gaze understanding 6 months prior and receptive vocabulary, even when correcting for age (see Fig. 4B and C). Taken together, our newly developed task shows connections to external variables and psychological constructs that are characteristic of measures of social cognition.

## General discussion

We have presented a new experimental paradigm to study gaze understanding across the lifespan. This paper contributes to methodological advances in developmental psychology in the following ways: first, we captured fine-grained individual differences in gaze understanding at different ages – from early childhood until adulthood. Individuals behaved consistently differently from one another (i.e., we found substantial variation between individuals across age groups). Second, our task showed satisfactory psychometric properties with respect to internal consistency and test–retest reliability estimates. Third, our new browser-based testing infrastructure ensures standardized, portable data collection at scale, both remotely as well as in person. In sum, the TANGO provides a step toward more robust and reliable research methods, especially with regard to measuring developmental change in a fundamental social-cognitive ability. The web app (https://ccp-odc.eva.mpg.de/tango-demo/) and its source code (https://github.com/ccp-eva/tango-demo) are freely accessible for use and modification.

Our continuous measure of children’s gaze understanding moves away from treating a social-cognitive ability as an all-or-nothing matter (e.g., dichotomous measures in pass/fail situations) toward an ability on a continuum (Beaudoin et al., 2020; Hughes & Devine, 2015). Identifying variability in social-cognitive abilities is vital for accurately quantifying developmental change, revealing relations between different aspects of cognition and children’s real-life social surroundings, and for meaningful comparisons across human cultures and across animal species. Dedicated measures of individual differences will help us to design meaningful interventions and progress in psychological theory building (Hedge et al., 2018).

Our continuous hedge version yields higher internal consistency estimates than the categorical box version. Both study versions exhibit high test–retest reliability, also when controlling for age. Therefore, when a sufficient number of trials is presented, the box version of the task can also yield reliable individual estimates (cf. Hughes et al. (2000); improved reliability through aggregation). When testing time is limited (and the number of trials might be low), we recommend using the continuous study version for higher internal consistency. However, the categorical box version demonstrates design features that might be preferable in some research contexts: for example, researchers could induce different levels of salience for each box. Our task could consequently be used to study bias, preferences, and diverse desires (e.g., matching the box appearance to some feature/behavioral characteristic of the agent).

In the split-half reliability calculations, the more accurately the statistical method represents the task structure, the higher the reliability estimates are. Therefore, we argue that future research should aim at implementing statistical analyses that mirror the complexity of the experimental design. Theoretically informed, computational cognitive models are a promising approach forward (Haines et al., 2020). Computational models take advantage of all available information and model variation between and within individuals in an even more fine-grained and psychologically interpretable manner. Computational frameworks could also be used to model performance and their underlying cognitive processes across tasks. With nested hierarchical models, we could assess the systematic relation between various social-cognitive abilities and recover potentially shared structures between cognitive processes (Bohn et al., 2023).

The TANGO fulfills several demands that were proposed by Schaafsma et al. (2015)’s wish list: it measures proficiency on a continuum, avoids floor and ceiling effects, measures variation across age ranges, shows satisfactory reliability estimates, and has a high face value.

In addition to the new task design itself, we designed a new testing infrastructure. The TANGO is presented as an interactive web app. This enables presentation across devices without any prior installation. Stimuli presentation is achieved through the use of SVGs. This has several advantages: the aspect ratio and stimulus quality are kept constant no matter which size the web browser displays. The cartoon-like presentation makes the task engaging for children and adults alike. Most importantly, we can dynamically modify the stimulus details (e.g., target positions) on a trial-by-trial basis. Presented agents, voice-over instructions, and objects can be easily adapted for future task modifications or specific linguistic and cultural settings.

The browser-based implementation allows for different data collection modes: participants can be tested in person with supervision or remotely at home. Test instructions are standardized, and with prior informed consent, the webcam records study participation. This allows us to scale up data collection: testing is flexible, fast, and requires no further experimenter training. We compared children participating in-person and supervised in kindergartens with children who participated remotely at home. Our results suggest a comparable developmental trajectory of gaze understanding in both samples. Children in the remote sample were slightly more precise. This effect was most pronounced in the 3-year-olds in the box version (for an analysis of the webcam recordings, see Supplements). Therefore, we recommend using a tablet for remote data collection. Children can click for themselves, and caregivers have less chance to interfere. The design choices of the infrastructure underline how our study design can act as a versatile framework for addressing further research questions on social-cognitive development.

With respect to validity, we found that performance in the TANGO was related to relevant external variables and cognitive measures. Family-level variables, capturing a child’s opportunity for social interaction, systematically influenced gaze understanding. Even though the effects were small and confidence intervals were wide, it is remarkable that we were able to detect relationships between this fundamental social-cognitive ability and very distant, real-life variables. In addition, we assessed the influence of gaze understanding on receptive vocabulary. We found a substantial relationship between the two variables, even when correcting for age. Taken together, this speaks to the validity of the TANGO.

### Limitations

First, we want to address the scope and interpretation of the TANGO. We believe that solving the task requires locating the attentional focus of an agent as the gaze cues the target location. This speaks to the face validity of the TANGO and its focus on an inherently social stimulus. However, we do not want to claim that the TANGO does not also recruit other, domain-general processes. For example, we believe that a considerable part of gaze understanding relies on vector-following: not just in our task but also in real life. From that perspective, gaze understanding could be seen as a particular case of vector-following that is learned and used in social interactions. Future research could assess how much variation of the gaze understanding task is shared with a physical vector-following task. In addition, computational cognitive models might prove helpful in defining children’s behavior on a process-level and disentangling parameters that influence task performance (e.g., spatial acuity).

Second, the influence of testing modality requires further attention. Remote data collection loosens the standardization of the experimental procedure, as we cannot prevent caregivers from interfering. Steering the child’s behavior becomes less possible when touchscreens are used, and the child can click on the screen directly. This is why we recommend using tablets for remote data collection. However, it should be noted that families’ access to technological devices varies, both across socio-environmental as well as cultural settings.

Third, the children in our sample live in an industrialized, urban Central-European context. It is unclear how our results would generalize to different socio-cultural contexts. A related limitation is that we did not collect demographical information on a participant-level and, instead, had to rely on a community-level description of the sample. This is important to keep in mind when gauging the generalizability of our new measure.

Finally, we utilized subtle gaze cues in order to increase difficulty and capture individual differences. However, in real-life settings, children could be more accustomed to a combination of head and eye orientation changes, and subtle gaze differences might be less common.

### Conclusions

**Fig. 5:**
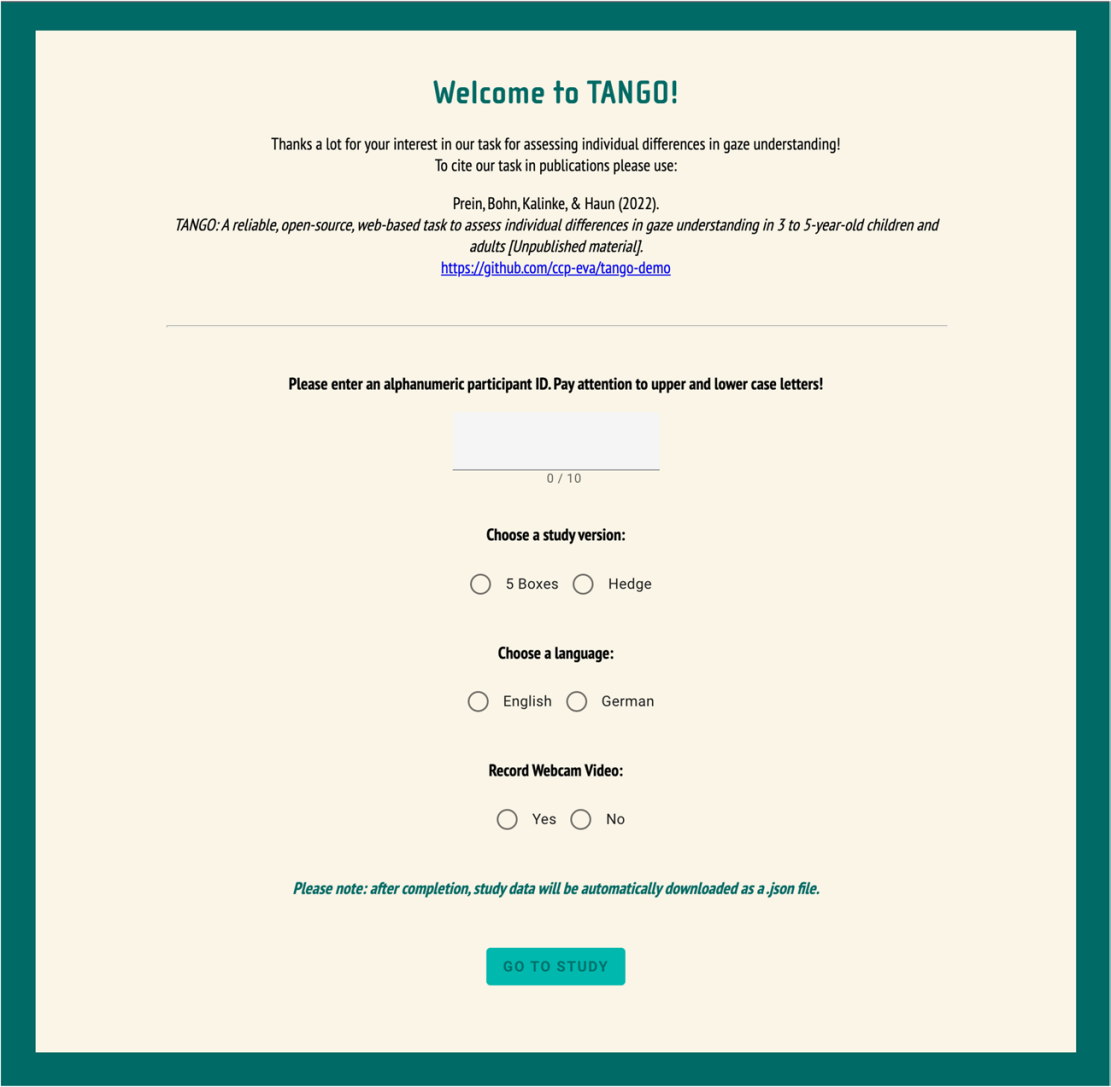
TANGO demo website. We want to highlight that researchers are welcome to use and modify our task according to their needs. The number of training and test trials and the number of boxes can be adjusted within the JavaScript code, while agents and targets can be exchanged within the HTML code

We have presented a new experimental paradigm to study gaze understanding across the lifespan. The TANGO captures individual differences and shows highly satisfactory psychometric properties with respect to internal consistency and test–retest reliability. The browser-based testing infrastructure allows for standardized, portable data collection at scale, both remotely as well as in person. Associations with social-environmental factors and language skills illustrate the validity of the task. Ultimately, this work shows a promising way forward toward more precise measures of cognitive development. The data sets and the analysis code are freely available in the associated online repository (https://github.com/ccp-eva/gazecues-methods). A demo version of the task is available at the following website (see Fig. 5): https://ccp-odc.eva.mpg.de/tango-demo/. The code base and respective assets can be accessed in the following repository: https://github.com/ccp-eva/tango-demo. These resources allow interested researchers to use, extend and adapt the task.

### Supplementary Information

Below is the link to the electronic supplementary material.

﻿Supplementary file1 (PDF 1.82 MB)

## Data Availability

The web application (https://ccp-odc.eva.mpg.de/tango-demo/) described here is open-source (https://github.com/ccp-eva/tango-demo). The data sets generated during and/or analyzed during the current study are available in the [gazecues-methods] repository (https://github.com/ccp-eva/gazecues-methods). All experiments were pre-registered (https://osf.io/zjhsc/).

## Footnotes

1 In the pre-registration (https://osf.io/snju6), we specified the following model structure: “All models will include a fixed effect of target centrality and age, a random intercept for ID and a random slope for trial number by ID. For both study versions, we will compare the above specified null models with a model including data source (live vs. online) as a fixed effect.” Or, in R model formula: R: performance ~ target_centrality + age + (1 | ID) + (1 + trial | ID). In this paper, we added symmetricPosition (synonymous to target_centrality) as a random slope because we expected that this item effect could vary between participants. To be better able to interpret trial number effects, we decided to include it as a fixed effect. Data collection mode (formerly named “data source”) proved as a meaningful predictor and was accordingly added to the model.

2 In an exploratory analysis, we coded parental behavior and environmental factors during remote unsupervised testing. We focused on the subsample with the greatest performance difference between data collection modes: the 3-year-olds in the box version of the task (*n* = 16). We reasoned that if parental interference cannot explain the greatest performance difference in our sample, the effects would be negligible in the remaining sample. Based on our model comparison, we conclude that there is no clear evidence of a stable effect of parental interference. See Supplements for further detail.

3 The assessment of internal consistency was not pre-registered and was included as an additional measure of reliability.

4 In the hedge version, we excluded one 3-year-old, one 5-year-old, and two adults from the test–retest analysis. The performance of the mentioned participants was 3 standard deviations above/below the mean of each sample. Including the two children yielded a *Pearson* correlation coefficient of *r* = 0.88. Including the two adults yielded a *Pearson* correlation coefficient of *r* = 0.73.
