# Supplementary material for: TANGO: A reliable, open-source, browser-based task to assess individual differences in gaze understanding in 3 to 5-year-old children and adults
Source: Behav Res Methods. 2023 Jul 10;56(3):2469–85. doi: 10.3758/s13428-023-02159-5 (PMC10991054; doi:10.3758/s13428-023-02159-5)
Supplement: Supplementary file 1 — ﻿Supplementary file1 (PDF 1.82 MB) [file 13428_2023_2159_MOESM1_ESM.pdf]

Supplemental Material for the manuscript 'TANGO: A reliable, open-source, browser-based task to assess individual differences in gaze understanding in 3 to 5-year-old children and adults'

Julia Prein[1], Manuel Bohn[1,2], Steven Kalinke[1], & Daniel B. M. Haun[1]

[1] Department of Comparative Cultural Psychology, Max Planck Institute for Evolutionary Anthropology, Leipzig, Germany

[2] Institute of Psychology, Leuphana University Lüneburg, Germany

Supplemental Material for the manuscript 'TANGO: A reliable, open-source, browser-based task to assess individual differences in gaze understanding in 3 to 5-year-old children and adults'

Effects of trial type and trial number

Children showed nearly perfect precision in the first training trial. As visual access to the target location decreased in the succeeding training trials, imprecision levels increased. Within test trials, children's imprecision levels did not vary as a function of trial number.

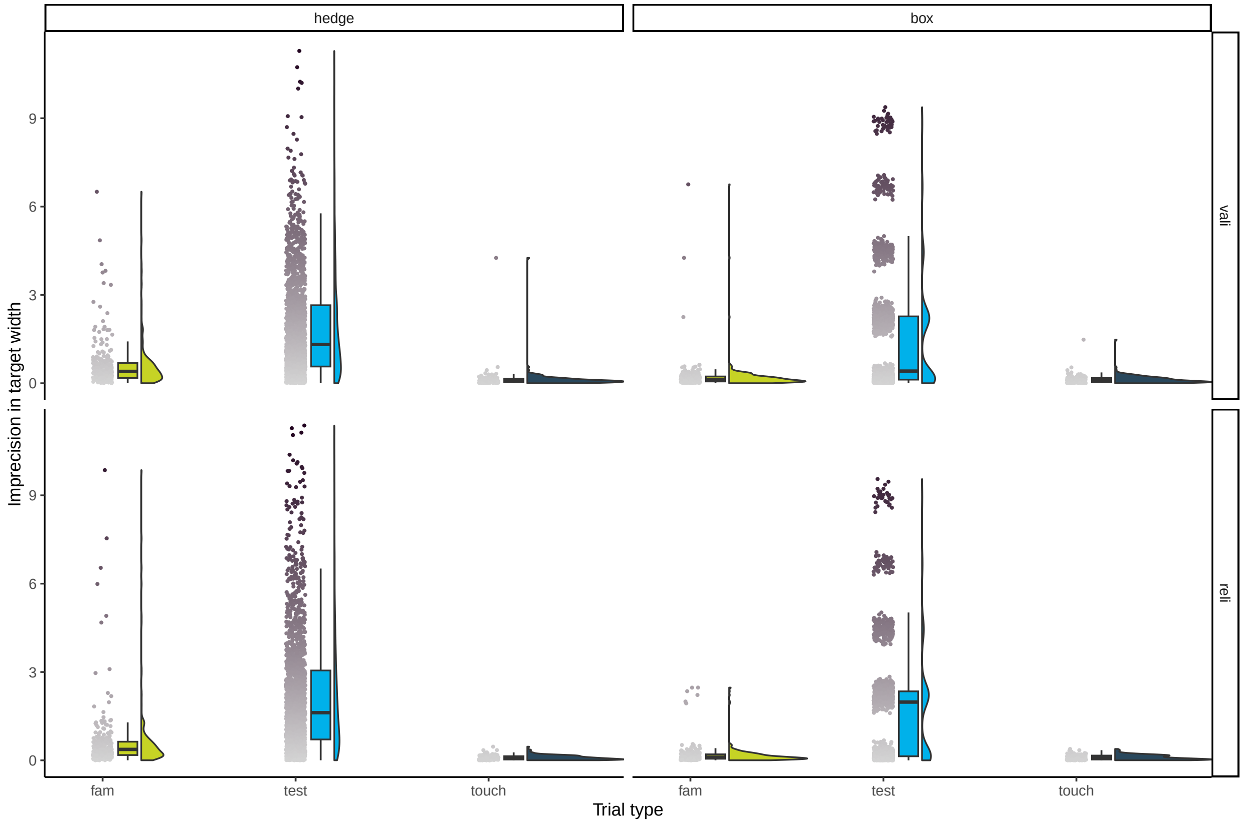

Figure 1. **Imprecision by trial type, split by study version and sample**. The x axis represents the trial type. The y axis represents imprecision, i.e., the absolute distance between the target's center and the participant's click. The unit of imprecision is counted in the width of the target, i.e., a participant with imprecision of 1 clicked one target width to the left or right of the true target center. Small dots show the imprecision for each subject in each trial. Boxplots (boxes represent first to third quartiles of data; vertical lines indicate the median; horizontal black lines display the range) and a half violin plot show the data distribution.

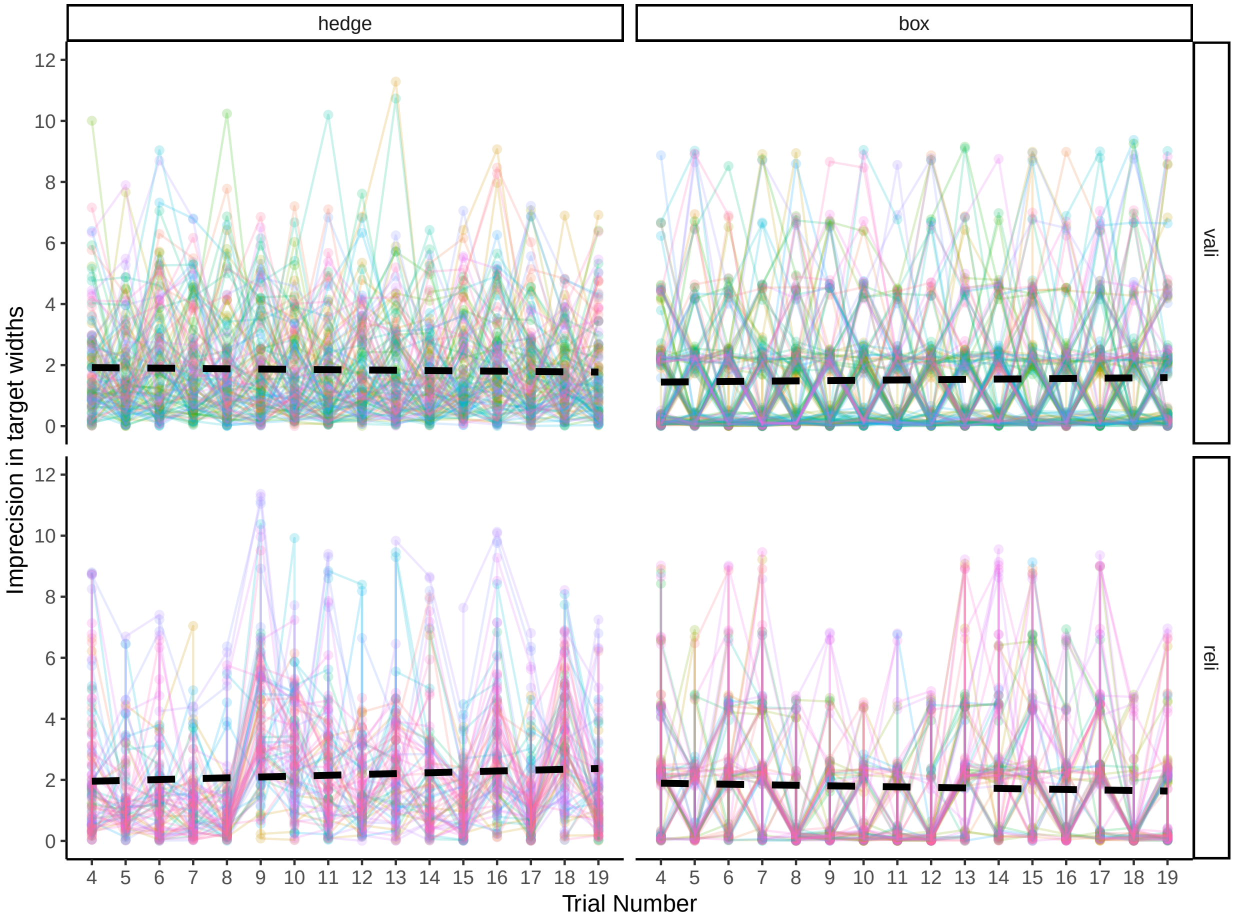

Figure 2. **Imprecision across test trials, split by study version and sample**. The x axis represents trial number. The y axis represents imprecision, i.e., the absolute distance between the target's center and the participant's click. The unit of imprecision is counted in the width of the target, i.e., a participant with imprecision of 1 clicked one target width to the left or right of the true target center. The black dashed regression lines show smooth conditional means based on linear models. Small colored dots show the imprecision for each subject in each trial. Colored lines connect the trials of each individual.

Webcam coding of the child sample

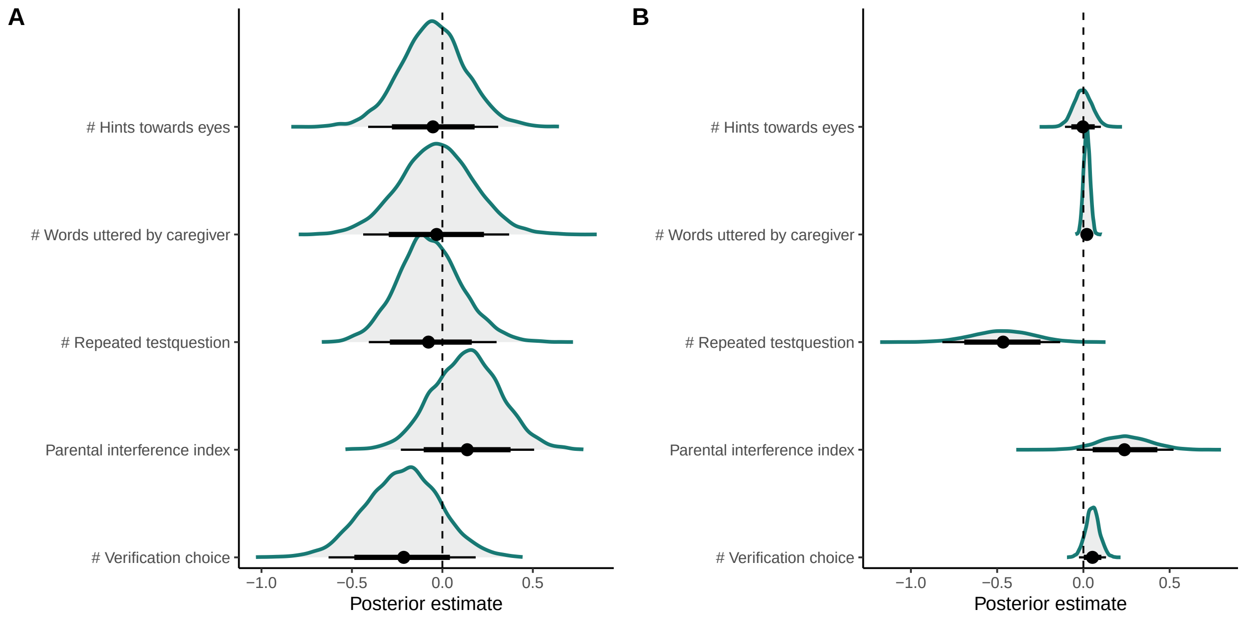

Figure 3. **Model comparison for exploratory webcam coding of parental interference**. Factors of parental interference and their influence on the probability of responding correctly. The graph shows the estimated density curves of a model's predictor coefficient. Models are ordered according to their WAIC scores in the trial-by-trial analysis, with the uppermost winning the model comparison. (A) Analysis on a trial-by-trial level. (B) Analysis on a subject level (i.e., average across trials per subject).

Comparing the performances of children across our two data collection modes, we found that children participating remotely were slightly more precise. This difference was especially prominent in younger participants in the box version of the task. It is conceivable that caregivers were especially prone to influence the behavior of younger children. In the box version, caregivers might have had more opportunities to interfere since they carried out the clicking for their children. In an exploratory analysis, we coded parental behavior and environmental factors during remote unsupervised testing. Due to the time consuming nature of hand coding videos frame by frame, we focused on the subsample with the greatest performance difference between data collection modes: the three-year-olds in the box version of the task (n = 0). We reasoned that if parental interference cannot explain the greatest performance difference in our sample, the effects

would be negligible in the remaining sample. A trial was defined as the time between two eye blinking sounds. We transcribed all utterances by parents and children and counted the words uttered by each. We then classified the utterances into several categories: question asked by child, repeated test questions by caregiver, hints towards agents (how many times the caregivers guided the child's attention to the agent), hints towards eyes (how many times the caregivers guided the child's attention to the agent's eyes), verification of choice (how many times the caregiver questioned or double checked the child's response), mentioning of screen (how many times the caregiver verbally guided the child's attention to the screen), pointing to screen (how many times the caregiver pointed towards the screen), positive & negative feedback, motivational statements, and incomprehensible utterances. In addition, we coded how many adults and children were present, whether a response click was obviously conducted by the caregiver themselves, and whether children took a break during the trial. We conducted a model comparison to estimate the effects of parental interference. Our null model explained the response behavior by age, while including random effects for subject and target position (model notation in `R: correct ~ age + symmetricPosition + (1 + symmetricPosition | subjID)`.

We compared this null model to models including the number of words uttered by the caregiver, number of repeated testquestions, verification of choice, or hints towards eyes as fixed effects. Furthermore, we calculated an parental interference index by summing up number of repeated testquestions, verification of choice, and hints towards eyes, with the sign matching the variable's direction of effect. Remaining variables that we coded for were not included since there was not enough variation and/or occurrences in our sample. We compared models using WAIC (widely applicable information criterion) scores and weights. As an indicator of out-of-sample predictive accuracy, lower WAIC scores stand for a better model fit. WAIC weights represent the probability that the model in question provides the best out-of-sample prediction compared to the other models. On the trial level, the model including the verification of choice as a main effect performed best: here, the less the

caregivers asked for children's responses again, the more likely children clicked on the correct box. Interestingly, the effect reversed on a subject level - possibly due to greater learning effects for the children that were most likely to click incorrectly in the beginning and then receiving most parental comments. On the subject level, the model including number of repeated test questions performed best: the more caregivers asked again where the target landed, the more likely children were to respond to the incorrect box. In all cases, however, ELPD difference scores were smaller than their standard errors. Similarly, 95% CI of the model estimates included zero and were rather wide. Therefore, we conclude that the effect of parental interference was negligable and could, most likely, be explained as described above.

Predictor	WAIC	SE_WAIC	Weight	ELPD_DIFF	SE_ELPD_DIFF
By trial - Null model	256.42	15.10	0.31	0.00	0.00
By trial - # Verification choice	256.57	15.46	0.29	-0.07	1.08
By trial - Parental interference index	258.08	15.38	0.14	-0.83	0.76
By trial - # Repeated testquestion	258.83	15.25	0.09	-1.20	0.61
By trial - # Hints eyes	258.88	15.30	0.09	-1.23	0.50
By trial - # Words uttered by caregiver	259.15	15.39	0.08	-1.37	0.36
By subject - # Repeated testquestion	83.45	10.20	0.82	0.00	0.00
By subject - Parental interference index	88.17	12.15	0.08	-2.36	3.55
By subject - Null model	89.36	11.28	0.04	-2.96	3.59
By subject - # Verification choice	89.94	12.01	0.03	-3.25	4.44
By subject - # Words uttered by caregiver	90.50	10.62	0.02	-3.52	4.19
By subject - # Hints eyes	93.02	11.84	0.01	-4.78	3.71

Reliability by age group

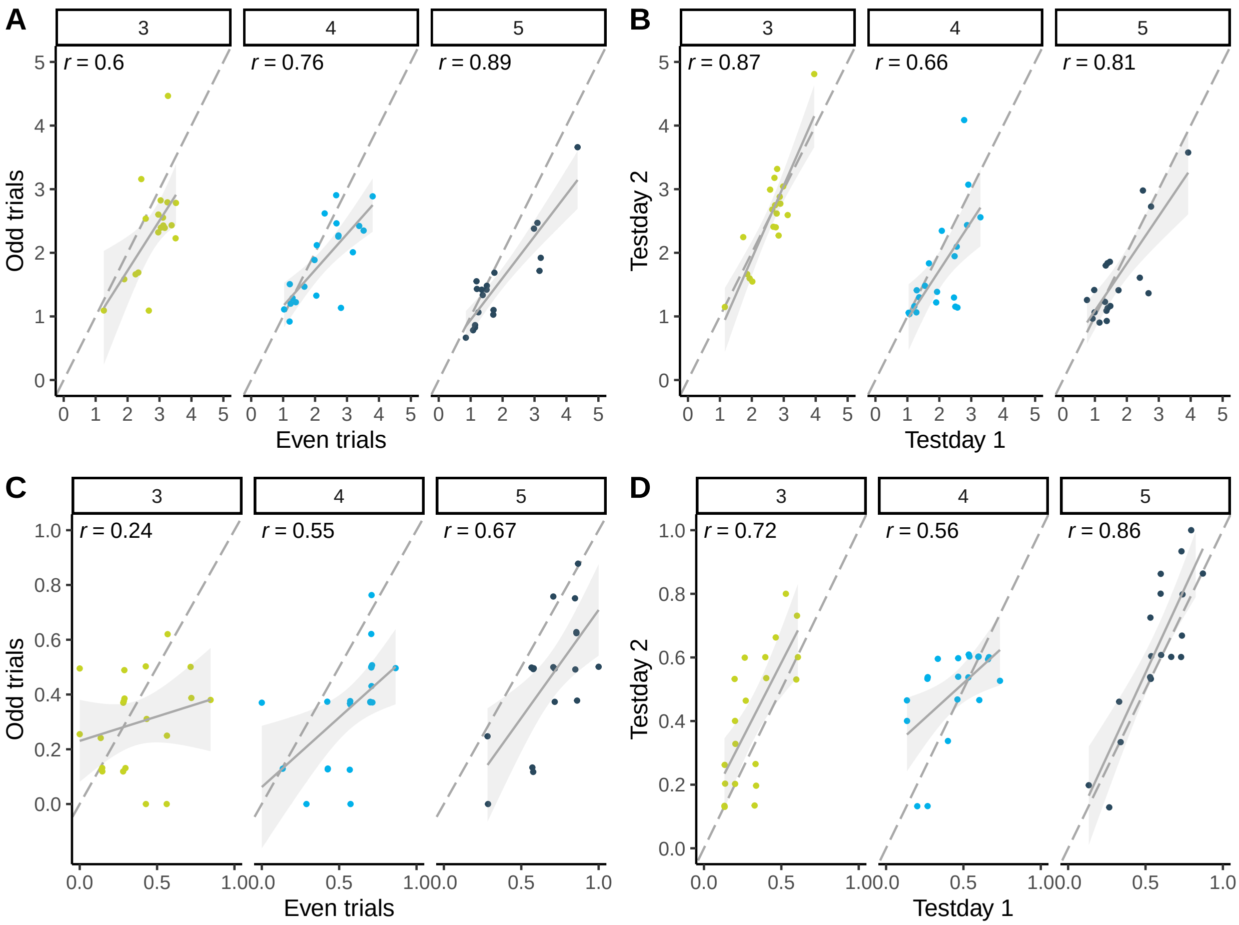

Figure 4. **Reliability split by age group**. (A) Internal consistency (odd-even split) in hedge child sample by age group. (B) Test-retest reliability in hedge child sample by age group. (C) Internal consistency (odd-even split) in box child sample by age group. (D) Test-retest reliability in box child sample by age group. For the hedge version, performance is measured as imprecision, i.e., the absolute distance between the target's center and the participant's click (averaged across trials). The unit of imprecision is counted in the width of the target, i.e., a participant with imprecision of 1 clicked on average one target width to the left or right of the true target center. For the box version, performance is measured as the proportion of correct responses, i.e., how many times the participant clicked on the box that contained the target. Regression lines with 95% CI show smooth conditional mean based on a linear model (generalized linear model for box version), with *Pearson*'s correlation coefficient *r*. Dots show the performance for each subject. The color of data points denotes age group.

Validity

Social-environmental factors

Participants. We included all children where families filled out a short demographic questionnaire. This subsample consisted of 109 children, including 30 3-year-olds (mean = 41.80 months, SD = 3.44, range = 36 - 47), 36 4-year-olds (mean = 54.19 months, SD = 3.08, range = 48 - 59), and 43 5-year-olds (mean = 66.28 months, SD = 3.62, range = 60 - 71).

Analysis. To estimate social environmental influences on gaze understanding, we fitted GLMMs predicting the task performance by each of our questionnaire variables, controlling for age (scaled), data collection mode (reference category: remote unsupervised) and study version (reference category: box version): `cor_tango | trials(n_tango) ~ age + datacollection + studyversion`). In order to combine data of our two study versions, we transformed continuous click responses from the hedge version into a discrete outcome. For the target position, we categorized two adjacent bins as one imaginary box. To measure participants' performance, we created imaginary box boundaries around the target's landing position and examined whether the participant's click response fell into this imaginary box. Across the two study versions, we could consequently model the number of participant's correct responses using a `Binomial` distribution. For model comparisons, we ran separate models, each with one of the following predictors as a fixed effects added to the null model: number of household members, number of children aged 0-18 in household, number of children aged 1-12 in household, hours spent in childcare each day, and age when subject entered childcare. In addition, we calculated three index scores. First, we calculated a sibling variety score according to Peterson (2000). Second, we implemented the modified version of Cassidy, Fineberg, Brown, and Perkins (2005). Third, based on our own data exploration, we calculated the amount of peer exposure determined as the number of siblings and the average hours spent in childcare. We compared the

models using WAIC (widely applicable information criterion) scores and weights (McElreath, 2020). As an indicator of out-of-sample predictive accuracy, lower WAIC scores stand for a better model fit. WAIC weights represent the probability that the model in question provides the best out-of-sample prediction compared to the other models.

Predictor	WAIC	SE_WAIC	Weight	ELPD_DIFF	SE_ELPD_DIFF
Age of childcare entry	571.54	28.09	0.72	0.00	0.00
Null model	575.80	30.43	0.09	-2.13	4.51
# Children in household aged 0-18	577.29	29.61	0.04	-2.88	4.70
Peer exposure index	577.90	30.20	0.03	-3.18	4.65
# Children in household aged 1-12	578.02	29.93	0.03	-3.24	4.69
Sibling variety score (Cassidy et al., 2005)	578.15	29.85	0.03	-3.31	4.66
Sibling variety score (Peterson, 2000)	578.21	29.84	0.03	-3.34	4.64
# Household members	578.30	30.32	0.02	-3.38	4.48
Average hours spent in childcare per day	579.04	30.66	0.02	-3.75	4.56

Results. Note that we did not find a great difference in WAIC scores between the compared models (see Supplements for WAIC scores and weights). The model estimates were all considerably smaller than estimates of age, study version and data collection, and all 95% CIs included zero. Nevertheless, a general pattern emerges: exposure to a more variable social environment positively influenced children's gaze understanding. The number of people and, more specifically, children, as well as the more diverse their age, the more likely children were to understand the agent's gaze cue. The only predictor resulting in a negative estimate was the age at which a participant entered childcare, i.e., the later a child entered, the better performance in the task. Effect sizes were probably influenced by the lack of variance in the predictors: variables like household size and number of siblings typically vary very little among German households (see distribution characteristics of predictor variables below).

	n	mean	sd	min	max	skew	kurtosis	se
Age of childcare entry in months	109	14.96	5.54	1.0	40	1.72	5.65	0.53
Average hours spent in childcare per day	109	7.50	0.92	3.5	9	-0.67	1.92	0.09
# Household members	109	3.72	0.92	2.0	7	0.30	0.49	0.09
# Children in household aged 0-18	109	0.83	0.79	0.0	4	0.87	1.09	0.08
# Children in household aged 1-12	109	0.69	0.74	0.0	4	1.23	2.67	0.07
Sibling variety score (Peterson, 2000)	109	0.60	0.56	0.0	2	0.23	-0.87	0.05
Sibling variety score (Cassidy et al., 2005)	109	0.63	0.54	0.0	2	0.18	-0.70	0.05

Receptive vocabulary

Participants. Our sample consisted of 117 children, including 11 3-year-olds (mean = 45.05 months, SD = 1.58, range = 43 - 47, 26 4-year-olds (mean = 53.17 months, SD = 3.01, range = 48 - 57), 32 5-year-olds (mean = 65.80 months, SD = 3.23, range = 61 - 71), 36 6-year-olds (mean = 78.36 months, SD = 3.26, range = 72 - 84), and 12 7-year-olds (mean = 88.11 months, SD = 1.96, range = 84 - 91).

Materials. We employed the oREV, an Item Response Theory based open receptive vocabulary task for 3 to 8-year-old children (Bohn et al., 2022). Similarly to the TANGO, the task was presented as an interactive web application (see Figure 5; live demo https://ccp-odc.eva.mpg.de/orev-demo/; source code https://github.com/ccp-eva/orev-demo).

Each trial presented four pictures: one target word alongside three distractors (1 phonological, 1 semantic, 1 unrelated distractor). A verbal prompt asked children to select one of the pictures (prompt: “Zeige mir [target word]”; engl.: “Show me [target word]”). The positioning of the target word and the three distractor types was counterbalanced across the four display positions (upper/lower and left/right corners). Children responded by clicking on one of the four pictures. The outcome measure consisted of the proportion of correct responses.

Procedure. Families received a personalized study link and completed the study at any location or time they wanted. Caregivers were asked to only provide technical support

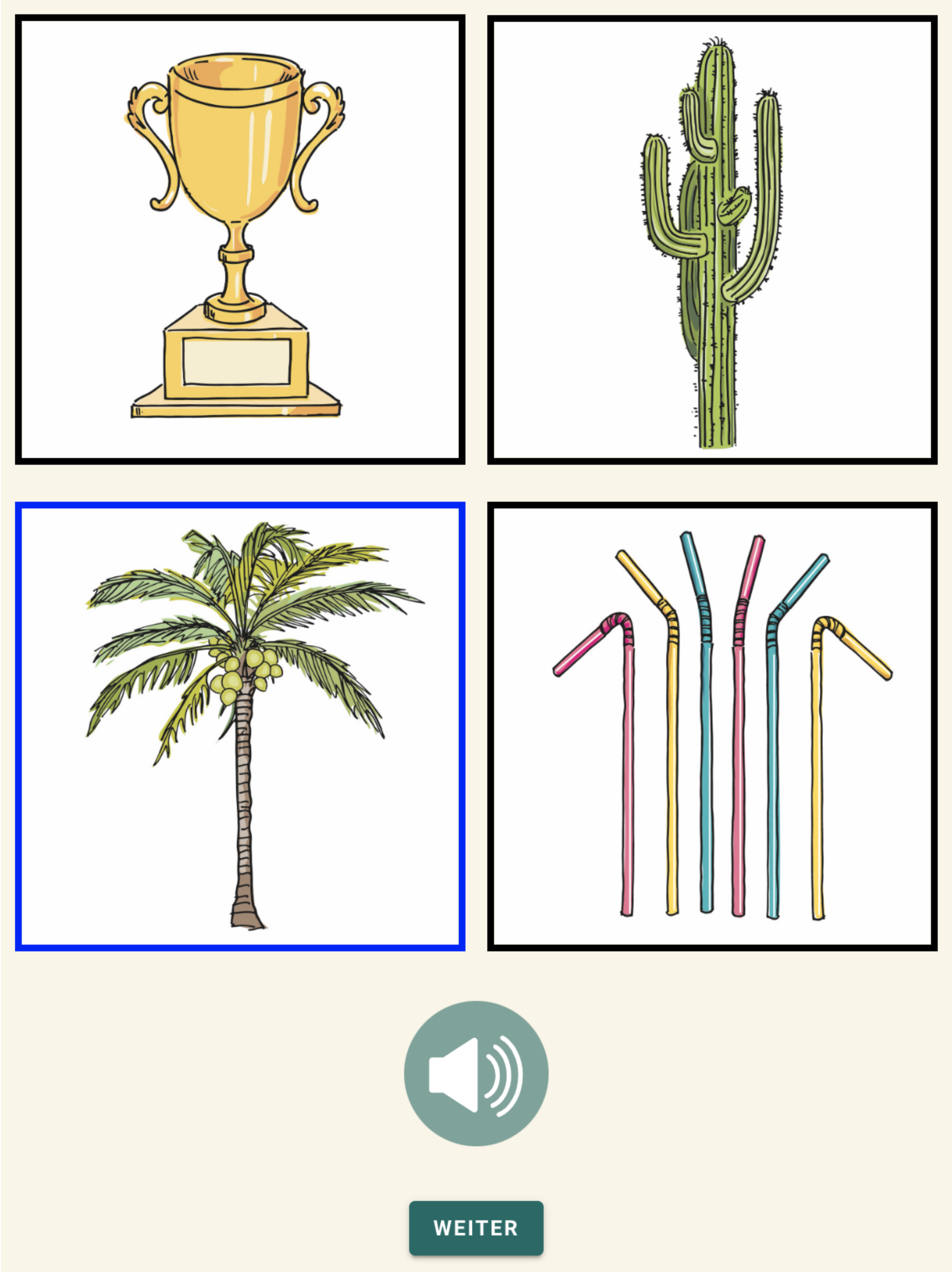

Figure 5. Setup of the oREV. On each trial, participants heard a word and were asked to select the corresponding picture. Verbal prompts could be replayed by pressing the loudspeaker button.

but no further help. Webcam recordings were done whenever consented and technically possible. We analyzed data of 20 trials per child. Children received the trials in two different orders. Performance across the two study orders did not differ. Hence, we decided to combine the data.

Analysis. To estimate the effect of gaze understanding on receptive vocabulary, we fitted a GLMM predicting the task performance by the gaze understanding score (scaled), controlling for age: `cor_clt | trials(n_clt) ~ age_centered + mean_gaze_centered`). We modelled the number of participant's correct responses using a `Binomial` distribution. In order to combine data of our two TANGO study versions, we transformed continuous click responses from the hedge version into a discrete outcome. After this transformation, we calculated a "TANGO score" as the proportion of correctly located targets. See Analysis section of Social-environmental factors for further detail.

Predictor	WAIC	SE_WAIC	Weight	ELPD_DIFF	SE_ELPD_DIFF
Age (scaled), TANGO score (scaled)	570.16	26.38	0.98	0.00	0.00
Age (scaled)	577.66	27.16	0.02	-3.75	4.16
Null model	635.72	28.70	0.00	-32.78	10.62

Results.

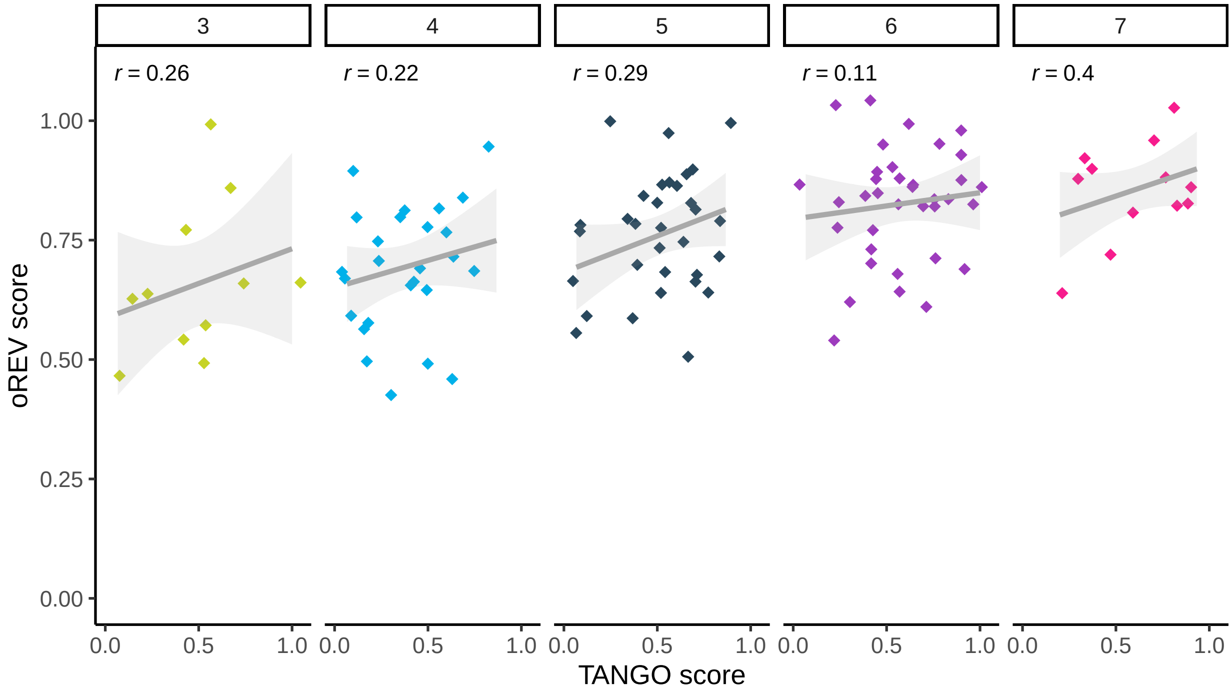

Figure 6. **Effect of gaze understanding on receptive vocabulary by age group**. Regression lines with 95% CI show smooth conditional mean based on a linear model (generalized linear model for box version), with *Pearson*'s correlation coefficient *r*. Diamonds show the performance for each subject. The color of data points denotes age group.

Additional information for the adult sample

currentResidence	studytype	n
United Kingdom	vali	47
South Africa	vali	8
Portugal	vali	6
Poland	vali	5
Ireland	vali	5
Italy	vali	4
Spain	vali	4
Netherlands	vali	4
Canada	vali	2

(continued)

currentResidence	studytype	n
Australia	vali	2
France	vali	2
Austria	vali	2
Norway	vali	1
DATA EXPIRED	vali	1
Finland	vali	1
Greece	vali	1
Germany	vali	1
United States	vali	1
Mexico	vali	1
South Africa	vali2	20
Portugal	vali2	11
United Kingdom	vali2	5
France	vali2	3
Italy	vali2	2
Spain	vali2	2
Poland	vali2	2
DATA EXPIRED	vali2	1
Finland	vali2	1
Czech Republic	vali2	1
Latvia	vali2	1
Switzerland	vali2	1
South Africa	reli	48

(continued)

currentResidence	studytype	n
United Kingdom	reli	19
United States	reli	14
Portugal	reli	10
Italy	reli	7
Canada	reli	6
Spain	reli	5
Ireland	reli	5
Poland	reli	4
Greece	reli	4
Netherlands	reli	3
CONSENT REVOKED	reli	3
Germany	reli	3
Australia	reli	2
France	reli	2
Czech Republic	reli	2
Hungary	reli	2
Chile	reli	1
Iceland	reli	1
New Zealand	reli	1

Recruitment

We recruited participants using the online participant recruitment service *Prolific* from the University of Oxford. *Prolific*'s subject pool consists of a mostly European and

US-american sample although subjects from all over the world are included. The recruitment platform realises ethical payment of participants, which requires researchers to pay participants a fixed minimum wage of £5.00 (around US$6.50 or €6.00) per hour. We decided to pay all participants the same fixed fee which was in relation to the estimated average time taken to complete the task. *Prolific* distributed our study link to potential participants, while the hosting of the online study was done by local servers in the Max Planck Institute for Evolutionary Anthropology, Leipzig. Therefore, study data was saved only on our internal servers, while *Prolific* provided demographic information of the participants. Participants' *Prolific* ID was forwarded to our study website using URL parameters. This way, we could match participant demographic data to our study data. The same technique was used to confirm study completion: we redirected participants from our study website back to the *Prolific* website using URL parameters. We used *Prolific*'s inbuilt prescreening filter to include only participants who were fluent in English and could therefore properly understand our written and oral study instructions.

Study 1 - Validation hedge version

The aim of Study 1 was to validate the hedge version of our gaze understanding task. The pre-registration can be found here: https://osf.io/r3bhn. We recruited participants online by advertising the study on *Prolific*.

50 adults participated in the study. One additional subject returned their submission, i.e., decided to leave the study early or withdrew their submission after study completion. Data collection took place in May 2021. Participants were compensated with £1.25 for completing the study. We estimated an average completion time of 6 minutes, resulting in an estimated hourly rate of £10.00. On average, participants took 05:56min to complete the study. Participants were required to complete the study on a tablet or desktop. Participation on mobile devices was disabled since the display would be too small and would harm click precision. It was indicated that the study required audio sound.

We stored *Prolific*'s internal demographic information, while not asking for additional personal information.

Study 2 - Validation box version

As in study 1, we recruited participants on *Prolific*, and employed the same methodology. However, this time we focussed on validating the box version of the task in an adult sample. Participants were presented with eight boxes in which the target could land. 50 adults participated in the study. One additional subject returned their submission, i.e., decided to leave the study early or withdrew their submission after study completion. Data collection took place in June 2021. Participants were compensated with £1.00 for completing the study. We estimated an average completion time of 6 minutes, resulting in an estimated hourly rate of £10.00. On average, participants took 04:43min to complete the study.

Study 3 - Reliability hedge version

In study 3 and 4, we assessed the test-retest reliability of our gaze understanding task in an adult sample. The pre-registration can be found here: https://osf.io/nu62m. We tested the same participants twice with a delay of two weeks. The testing conditions were as specified in Study 1 and 2. However, the target locations as well as the succession of animals and target colors was randomized once. Each participant then received the same fixed randomized order of target location, animal, and target color. Participants received 30 test trials without voice-over description, so that each of the ten bins occurred exactly three times.

In addition to the aforementioned prescreening settings, we used a whitelist. *Prolific* has a so-called *custom allowlist prescreening filter* where one can enter the *Prolific* IDs of participants who completed a previous study. Only these subjects are then invited to

participate in a study. This way, repeated measurements can be implemented, collecting data from the same subjects at different points in time.

In a first round, 60 participants took part on the first testday. Additional two subjects returned their submission, i.e., decided to leave the study early or withdrew their submission after study completion. One additional participant timed out, i.e., did not finish the survey within the allowed maximum time. The maximum time is calculated by *Prolific*, based on the estimated average completion time. For this study, the maximum time amounted to 41 minutes. For the first testday, participants were compensated with £1.25. We estimated an average completion time of 9 minutes, resulting in an estimated hourly rate of £8.33. On average, participants took 07:11min to complete the first part.

Of the 60 participants that completed testday 1, 41 subjects finished testday 2. One additional participant timed out, i.e., did not finish the survey within the allowed maximum time. Participants were compensated with £1.50 for completing the second part of the study. We estimated an average completion time of 9 minutes, resulting in an estimated hourly rate of £10. On average, participants took 06:36min to complete the second part of the study.

Since we aimed for a minimum sample size of 60 subjects participating on both testdays, we reran the first testday with additional 50 participants. Additional seven subjects returned their submission, i.e., decided to leave the study early or withdrew their submission after study completion. Two additional participants timed out, i.e., did not finish the survey within the allowed maximum time. Again, participants were compensated with £1.25 for completing the first part of the study (estimated average completion time 9 minutes, estimated hourly rate of £8.33). On average, participants took 06:51min to complete the first part.

Of the additional 50 participants that completed testday 1, 29 subjects finished testday 2. Again, participants were compensated with £1.50 for completing the second

part of the study (estimated average completion time 9 minutes, estimated hourly rate of £10). On average, participants took 06:26min to complete the second part of the study.

Study 4 - Reliability box version

As in study 3, we recruited participants on *Prolific*, and employed the same methodology. However, this time participants were presented with the box version of the task. Participants received 32 test trials without voice-over description, so that each of the eight boxes occurred exactly four times. As in study 2, we employed eight boxes in which the target could land.

In a first round, 60 participants took part on the first testday. Additional five subjects returned their submission, i.e., decided to leave the study early or withdrew their submission after study completion. For the first testday, participants were compensated with £1.25. We estimated an average completion time of 9 minutes, resulting in an estimated hourly rate of £8.33. On average, participants took 07:33min to complete the first part.

Of the 60 participants that completed testday 1, 41 subjects finished testday 2. Participants were compensated with £1.50 for completing the second part of the study. We estimated an average completion time of 9 minutes, resulting in an estimated hourly rate of £10. On average, participants took 07:50min to complete the second part of the study.

Since we aimed for a minimum sample size of 60 subjects participating on both testdays, we reran the first testday with additional 50 participants. Additional eight subjects returned their submission, i.e., decided to leave the study early or withdrew their submission after study completion. One additional participant timed out, i.e., did not finish the survey within the allowed maximum time. Again, participants were compensated with £1.25 for completing the first part of the study (estimated average completion time 9 minutes, estimated hourly rate of £8.33). On average, participants took 07:37min to

complete the first part.

Of the additional 50 participants that completed testday 1, 28 subjects finished testday 2. Additional three subjects returned their submission, i.e., decided to leave the study early or withdrew their submission after study completion. One additional participant timed out, i.e., did not finish the survey within the allowed maximum time. Again, participants were compensated with £1.50 for completing the second part of the study (estimated average completion time 9 minutes, estimated hourly rate of £10). On average, participants took 06:30min to complete the second part of the study.

Instructions and voice-over descriptions

This is the content of our audio recordings that were played as instructions and during voice-over trials.

**Timeline**	**German**	**English**	**Filename**
**welcome**	Hallo! Schön, dass du da bist. Wir spielen jetzt das Ballon-Spiel! Siehst du die Tiere auf dem Bild da? Wir möchten gleich zusammen mit den Tieren mit einem Ballon spielen. Was genau passiert, erklären wir dir jetzt ganz in Ruhe.	Hello! Great that you're here. We'll now play a balloon game. Can you see the animals in the picture over there? We want to play together with the animals using the balloon. We'll now talk you through exactly what will happen.	welcome.mp3

**touch**	Schau mal, da steht ein Tier im Fenster. Und siehst du den Ballon da? Der Ballon fällt immer runter und landet auf dem Boden. Und du musst ihn dann finden. Das Tier hilft Dir und schaut immer den Ballon an.	Look, an animal is standing in the window. And can you see the balloon over there? The balloon always falls down and lands on the ground. And you have to find it! The animal helps you and always looks at the balloon.	touch-1.mp3
	Wo ist der Ballon? Drück auf den Ballon!	Where is the balloon? Click on the balloon!	prompt-touch-long.mp3

**fam - HEDGE**	Klasse, das war super! Jetzt spielen wir weiter. Siehst du wieder das Tier und den Ballon da? Der Ballon fällt wieder runter. Diesmal fällt er hinter eine Hecke. Du musst ihn wieder finden. Das Tier hilft dir und schaut immer den Ballon an.	Perfect, that was great! Now, we'll continue playing. Can you see the animal and the balloon again? The balloon will fall down again. This time, it will fall behind a hedge. And you have to find it! The animal helps you and looks at the balloon.	fam-hedge-1.mp3
	Wo ist der Ballon? Drücke auf die Hecke - wo der Ballon ist.	Where is the balloon? On the hedge, click where the balloon is.	prompt-hedge-long.mp3

**fam - BOX**	Klasse, das war super! Jetzt spielen wir weiter. Siehst du wieder das Tier und den Ballon da? Der Ballon fällt wieder runter. Diesmal fällt er in eine Kiste. Du musst ihn wieder finden. Das Tier hilft dir und schaut immer den Ballon an.	Perfect, that was great! Now, we'll continue playing. Can you see the animal and the balloon again? The balloon falls down again. This time, it falls into a box. And you have to find it! The animal helps you and looks at the balloon.	fam-box-1.mp3
	Wo ist der Ballon? Drücke auf die Kiste mit dem Ballon.	Where is the balloon? Click on the box with the balloon.	prompt-box-long.mp3
**test - HEDGE**	Klasse , das hast du toll gemacht! Nun spielen wir weiter. Da sind wieder der Ballon, das Tier und die Hecke. Die Hecke wächst jetzt hoch.	Nice, good job! Now, we'll continue playing. There is the balloon, the animal and the hedge. The hedge is growing a bit now.	test-hedge-1.mp3

	Der Ballon ist nun hinter der Hecke. Du kannst das nicht sehen - das Tier aber! Jetzt fällt der Ballon auf den Boden und du musst ihn wieder finden. Denk dran - das Tier schaut immer den Ballon an.	The balloon is behind the hedge now. You can't see it - but the animal can! The balloon falls to the ground and you have to find it. Remember - the animal always looks at the balloon!	test-hedge-2.mp3
	Dann schrumpft die Hecke. Drücke auf die Hecke - wo der Ballon ist.	Now, the hedge is shrinking. On the hedge, click where the balloon is.	test-hedge-3.mp3
**test - BOX**	Klasse , das hast du toll gemacht! Nun spielen wir weiter. Da sind wieder der Ballon, das Tier und die Kisten. Jetzt wächst eine Hecke hoch.	Nice, good job! Now, we'll continue playing. There is the balloon and the animal. Now, a hedge is growing.	test-box-1.mp3

	Der Ballon ist nun hinter der Hecke. Du kannst das nicht sehen - das Tier aber! Jetzt fällt der Ballon in eine Kiste und du musst ihn wieder finden. Denk dran - das Tier schaut immer den Ballon an.	The balloon is behind the hedge now. You can't see it - but the animal can! The balloon falls into a box and you have to find it. Remember - the animal always looks at the balloon!	test-box-2.mp3
	Dann schrumpft die Hecke. Drücke auf die Kiste mit dem Ballon.	Now, the hedge is shrinking. Click on the box with the balloon.	test-box-3.mp3
**goodbye**	Geschafft! Die Tiere sind schon ganz glücklich vom Spielen! Vielen Dank für deine Hilfe! Bis zum nächsten Mal und liebe Grüße vom Schwein, Affen und Schaf	The animals are super happy after playing. Thanks a lot for your help! See you soon and goodbye from the pig, monkey and sheep	goodbye.mp3
**general prompt**	Wo ist der Ballon?	Where is the balloon?	prompt-general.mp3

**touch - no response**	Drück auf den Ballon!	Click on the balloon!	prompt-touch.mp3
**hedge - no response**	Drücke auf die Hecke - wo der Ballon ist!	On the hedge, click where the balloon is!	prompt-hedge.mp3
**box - no response**	Drücke auf die Kiste mit dem Ballon!	Click on the box with the balloon!	prompt-box.mp3
**landing sound of balloon**	-	-	balloon-lands.mp3
**sound of blinking eyes**	-	-	blink.mp3
**sound for target click**	-	-	positive-feedback.mp3

References

Bohn, M., Prein, J. C., Koch, T., Bee, R. M., Delikaya, B., Haun, D. B. M., & Gagarina, N. (2022). *oREV: An Item Response Theory based open receptive vocabulary task for 3 to 8-year-old children* [Preprint]. PsyArXiv. https://doi.org/10.31234/osf.io/4z86w

Cassidy, K. W., Fineberg, D. S., Brown, K., & Perkins, A. (2005). Theory of Mind May Be Contagious, but You Don't Catch It from Your Twin. *Child Development*, *76*(1), 97–106. Retrieved from https://www.jstor.org/stable/3696711

McElreath, R. (2020). *Statistical rethinking: A Bayesian Course with Examples in R and Stan* (Second). Chapman and Hall/CRC.

Peterson. (2000). Kindred spirits: Influences of siblings' perspectives on theory of mind. *Cognitive Development*, *15*(4), 435–455. https://doi.org/10.1016/S0885-2014(01)00040-5
